# Multicentre evaluation of the pharmacological management of women with bipolar disorder in the perinatal period

**DOI:** 10.1192/bjo.2021.455

**Published:** 2021-06-18

**Authors:** Marisa Casanova Dias, Ian Jones, Angelika Wieck

**Affiliations:** 1 Divison of Psychological Medicine and Clinical Neuroscience, Cardiff University; 2 Greater Manchester Mental Health NHS Foundation Trust

## Abstract

**Aims:**

The pharmacological management of women with bipolar disorder in the perinatal period is challenging. This population has a high recurrence rate, but some medications can be a concern in pregnancy and breastfeeding. Little is known about prescribing practices in perinatal services, and the impact of medication on recurrence rates.

The aims of this study are: 1) to describe the use of medication in women with bipolar disorder in the perinatal period and 2) to evaluate the impact of medication on the rate of postpartum recurrence.

**Method:**

Clinical data were collected from pregnant women with a diagnosis of bipolar disorder in the nine participating centres in the UK and who were not experiencing an episode of illness entering the postpartum period. Using a proforma, data were collected for the period between conception and three months postpartum: sociodemographic, reproductive, the severity of illness, medication and recurrence.

Data were analysed for association using χ2 tests and logistic regression.

**Result:**

In this sample of 167 women, 91 (55%) were taking medication at delivery: 62 (37%) antipsychotics, 41 (25%) antidepressants, and 25 (15%) mood stabilisers. In 12 cases medication was reduced before delivery. Of those who were taking medication at delivery six decreased of stopped after delivery and one increased the dose. 42% of women in this sample experienced a recurrence, with 30% of the sample experiencing a manic/psychotic episode. There was no significant association between taking medication and recurrence χ 2(1) = 0.07, p = 0.79. There continued to be no association in a multivariable analysis when adjusted for parity, severity (previous admissions, age at first treatment, bipolar subtype), type of medication during pregnancy and immediate postpartum changes aOR 0.33 95%CI [0.03; 3.40], p = 0.35.

**Conclusion:**

A high number of women with bipolar disorder are taking medication before delivery and in the majority, antipsychotics are prescribed. The postnatal recurrence rate in both medicated and unmedicated women is high. These results are in line with existing literature. Further work is needed in larger samples to provide clinical guidance for women and their clinicians.

